# Ligand binding modulates the structural dynamics and activity of urokinase-type plasminogen activator: A possible mechanism of plasminogen activation

**DOI:** 10.1371/journal.pone.0192661

**Published:** 2018-02-08

**Authors:** Tobias Kromann-Hansen, Eva Louise Lange, Ida K. Lund, Gunilla Høyer-Hansen, Peter A. Andreasen, Elizabeth A. Komives

**Affiliations:** 1 Department of Chemistry and Biochemistry, University of California at San Diego, La Jolla, California, United States; 2 Department of Molecular Biology and Genetics, Aarhus University, Aarhus, Denmark; 3 The Finsen Laboratory, Rigshospitalet, Copenhagen, Denmark; 4 Biotech Research & Innovation Centre (BRIC), University of Copenhagen, Copenhagen, Denmark; Russian Academy of Medical Sciences, RUSSIAN FEDERATION

## Abstract

The catalytic activity of trypsin-like serine proteases is in many cases regulated by conformational changes initiated by binding of physiological modulators to exosites located distantly from the active site. A trypsin-like serine protease of particular interest is urokinase-type plasminogen activator (uPA), which is involved in extracellular tissue remodeling processes. Herein, we used hydrogen/deuterium exchange mass spectrometry (HDXMS) to study regulation of activity in the catalytic domain of the murine version of uPA (muPA) by two muPA specific monoclonal antibodies. Using a truncated muPA variant (muPA^16-243^), containing the catalytic domain only, we show that the two monoclonal antibodies, despite binding to an overlapping epitope in the 37s and 70s loops of muPA^16-243^, stabilize distinct muPA^16-243^ conformations. Whereas the inhibitory antibody, mU1 was found to increase the conformational flexibility of muPA^16-243^, the stimulatory antibody, mU3, decreased muPA^16-243^ conformational flexibility. Furthermore, the HDXMS data unveil the existence of a pathway connecting the 70s loop to the active site region. Using alanine scanning mutagenesis, we further identify the 70s loop as an important exosite for the activation of the physiological uPA substrate plasminogen. Thus, the data presented here reveal important information about dynamics in uPA by demonstrating how various ligands can modulate uPA activity by mediating long-range conformational changes. Moreover, the results provide a possible mechanism of plasminogen activation.

## Introduction

Urokinase-type plasminogen activator (uPA) is a trypsin-like serine protease that plays a pivotal role in fibrinolysis in the extracellular space as initiator of a protein cascade eventually leading to generation of another trypsin-serine protease, plasmin. Plasmin, in turn, acts directly to degrade fibrin and indirectly, and relatively nonspecifically, to activate matrix-metalloproteases which then degrade collagen [1]. Under normal physiological conditions, the proteolytic activity of uPA is involved in processes such as wound healing. However, under abnormal pathophysiological conditions, the activity of uPA is implicated in tissue remodeling in several diseases including rheumatoid arthritis, progression of cancer and atherosclerosis [2–6].

uPA is a three-domain protein consisting of a receptor binding domain (epidermal growth factor like domain), a kringle domain, and a catalytic serine protease domain. The serine protease domain adopts the typical double *β*-barrel chymotrypsin-like fold containing 6 *β*-strands in each *β*-barrel (the N-terminal *β*-barrel (*β*1- *β*6) and the C-terminal *β*-barrel (*β*7- *β*12)) flanked by 3 α-helices (α1-α3) and 11 connecting loops [7]. uPA is synthesized as a low-activity single-chain zymogen that is processed by plasmin to a two-chain form. Plasmin cleavage of single-chain uPA liberates a new N-terminus (Ile16) that becomes inserted into a hydrophobic binding cleft referred to as the activation pocket. This results in ordering of several surface-exposed loops, including the N-terminal activation loop (residues 16–21), the 140s loop (residues 142–152), the 180s loop (residues 184–194) and the 220s loop (residues 216–223) [8]. As a result of ordering these loops, the active site of uPA (His57, Asp102 and Ser195) including the primary S1 substrate-binding pocket (Asp189) and the oxyanion hole defined by the main chain amides of Gly193 and Ser195, adopts a fully-catalytically active conformation [9].

Trypsin-like serine proteases have very dynamic structures and exist even in their activated two-chain form as conformational ensembles constantly interconverting between active and inactive states [10–14]. We have recently determined X-ray crystal structures of the murine version of uPA (muPA) in an active and an inactive conformation [15]. The active conformation displayed a catalytically competent state with the N-terminal Ile16 inserted into the activation pocket and with a correctly assembled oxyanion hole and S1 specificity pocket. The inactive conformation displayed a catalytically incompetent state with the N-terminal Ile16 solvent-exposed and with disintegrated catalytic machinery.

We have previously identified an exosite in the 37s and 70s loops in the N-terminal β-barrel of murine uPA (muPA), and showed how the binding of two monoclonal antibodies to this site affected the activity of muPA differently. Whereas one of the antibodies, mU1, blocked muPA-mediated plasminogen activation and plasmin-mediated single-chain muPA activation, the other antibody, mU3, was only blocking muPA-mediated plasminogen activation [16, 17]. Moreover, mU1 but not mU3 was found to inhibit muPA-dependent processes *in vivo [18, 19]*. It was further shown that mU1 but not mU3 competitively inhibits muPA hydrolysis of a small tri-peptide chromogenic substrate [16, 17]. These studies also revealed that mU1 remained inhibitory towards a truncated muPA variant that only contained the catalytic serine protease domain (muPA^16-243^). Surprisingly, it was further shown that mU3 stimulates the catalytic activity of muPA^16-243^ by 2-fold (16, 17).

In this study, we sought to investigate the molecular mechanisms behind the differences in functional effects of mU1 and mU3 to the catalytic activity of muPA^16-243^ by evaluating the conformational flexibility of muPA^16-243^ by hydrogen/deuterium exchange mass spectrometry (HDXMS) in the absence or presence of mU1, mU3 or the active site binding ligand Glu-Gly-Arg-chloromethylketone (EGR-cmk). The results reveal that mU1 and mU3, despite sharing an overlapping binding epitope, stabilize two widely different muPA^16-243^ solution conformations. Whereas mU1-bound muPA^16-243^ displayed increased conformation flexibility, mU3-bound and EGR-cmk-bound muPA^16-243^ displayed decreased conformational flexibility in surface-exposed loops surrounding the active site. As the HDXMS results reveals a functional link between the 70s loop and the active site region, we next investigated if the 70s loop serves as an exosite during cleavage of the physiological uPA substrate plasminogen. A kinetic analysis of plasminogen activation by alanine-substituted full-length muPA mutants revealed that residues Glu73 and Ser74 may be important during plasminogen activation as K_M_ was significantly increased for the muPA E73A and S74A mutants. Taken together, our data reveal important mechanistic insight into how long-range conformational changes are mediated from the 37s and 70s loop to the active site region in muPA thereby providing a rationale for a possible plasminogen activation mechanism.

## Materials and methods

### Generation of mU1, mU3, FabmU1 and FabmU3

The generation and characterization of mU1 and mU3 were described previously [16–18]. For digestion of full-length IgGs, mU1 and mU3 (10 mg) was dialyzed against a cleavage buffer containing 0.1 M Na_2_HPO_4_ and 0.1 M KH_2_PO_4_ at pH 7.3. After dialysis, the IgGs were cleaved by incubation with 15 mM cysteine, 2 mM EDTA and 100 μg papain for 16 h at 37°C in the dark. The cleavage reactions where stopped by adding iodoacetamide to 20 mM. The generated Fab fragments were purified by Protein A affinity chromatography (Ge Healthcare) by using 50 mM Tris pH 7 as wash buffer and 0.1 M citric acid pH 3.0 as elution buffer. The purity of the generated Fab fragments was evaluated by SDS-PAGE analysis.

### Expression and purification of muPA

Full-length muPA and site-directed alanine mutants were produced in mammalian HEK293 6E suspension cells as previously described [16, 20]. The catalytic serine protease domain of muPA (muPA^16-243^) was produced by subcloning cDNA encoding amino acids Gly2 through Gly243 with a C122A mutation into a T7 derived expression vector with six histidines at the N-terminus, and expressed as inclusion bodies in *E*.*coli* BL21(DE3) (Novagen). For refolding of muPA^16-243^ *E*.*coli* cells were resuspended in sonication buffer (50 mM Tris pH 8.0; 0.5M NaCl; 10% (v/v) glycerol; 1 mM *beta*-mercaptoethanol; 1 mM EDTA) and sonicated on ice (pulses of 0.8, amplitude 100). The lysed cells were centrifuged at 10,000 RPM, 4°C for 10 min and the inclusion bodies were washed in sonication buffer supplemented with 1% (v/v) Triton X-100. Two additional washes were performed with 0.25% (v/v) and 0% (v/v) Triton X-100 respectively. Finally the inclusion bodies was resuspended in denaturation buffer (50 mM Tris pH 8.0; 100 mM NaCl; 10 mM β-mercaptoethanol; 6 M urea; 1 mM EDTA) and denatured by slow stirring at 4°C. Next the protein concentration was adjusted to below 0.2 mg/mL in denaturation buffer and dialysed against 10 L of refolding buffer (50 mM Tris pH 8.0; 1 mM β-mercaptoethanol; 3 M urea; 10% glycerol) at 4°C for 22 h. Urea was removed by dialysis against 2x10 L of buffer containing 50 mM Tris pH 8.0 and 10% glycerol at 4°C for 22 h. After solubilization and refolding muPA was subsequently captured on nickel-sepharose and eluted in 50 mM Bicine pH 8.0; 500 mM NaCl and 400 mM imidazole and dialyzed extensively against PBS (10 mM Na_2_HPO_4_; 1.8 mM KH_2_PO_4_; 2.7 mM KCl; 137 mM NaCl; pH 7.4). The protein concentration was adjusted to 0.5 mg/mL and incubated with 2.5 μg/mL plasmin at 22°C for 22h. The incubation with plasmin ensured correct cleavage between Lys15 and Ile16 to generate the catalytic domain. Plasmin was removed by passing the sample over a CNBr (GE Healthcare) activated aprotinin sepharose column. To remove non-activated or misfolded protein benzamidine-sepharose (GE Healthcare) chromatography was applied. Finally the muPA^16-243^ active catalytic domain was purified by size-exclusion chromatography on a Superdex 75 equilibrated with PBS supplemented with 300 mM NaCl. Protein purity was verified by SDS-PAGE analysis.

### Hydrogen deuterium exchange mass spectrometry

EGR-cmk-bound muPA^16-243^ was prepared by incubating muPA^16-243^ with 10-fold molar excess of EGR-cmk (Bachem, USA) for 1 h at 22°C in PBS. Excess EGR-cmk was removed by dialyzing against 2 L PBS at 4°C for 16 h. The Fab- muPA^16-243^ complexes were generated by incubating 5 μM muPA^16-243^ with 17 μM FabmU1 or 10 μM FabmU3 for 15 min at 22°C. The concentrations of FabmU1 or FabmU3 were estimated based on the equilibrium binding constants between the full-length IgGs and the catalytic domain of muPA as reported in [16, 17] in order to obtain 99% bound muPA^16-243^ in the samples.

The HDXMS analysis was performed using a Waters Synapt G2Si System with HDX technology (Waters Coorporation) and a LEAP HDX PAL autosampler (Leap Technologies). 10 mL D_2_O buffer was prepared by vacuum centrifugation (SpeedVac SC 100, Savant) of 1 mL 10xPBS and resuspending in 10 mL 99.96% D_2_O immediately before use. For deuterium exchange the proteins was mixed with D_2_O buffer an allowed to incubate for 0, 30 s, 1 min, 2 min or 5 min at 25°C. The reactions were quenched at pH 2.6 (3 M Guanidine HCl, 0.1% (v/v) formic acid and 250 mM TCEP) for 1 min at 1°C and injected on an in-line pepsin column (Pierce, Inc.). The resulting peptides were captured on a BEH C18 Vanguard pre-column, separated by analytical chromatography (Acquity UPLC BEH C18, 1.7 μm, 1.0x50 mm, Waters Corporation) using a 7–85% (v/v) acetonitrile gradient in 0.1% (v/v) formic acid over 7.5 min, and electrosprayed into the Waters Synapt G2Si quadrupole time-of-flight mass spectrometer. The mass spectrometer was set to collect data in the Mobility, ESI+ mode; mass acquisition range of 200–2000 (m/z); scan time 0.4s. Continuous lock mass correction was accomplished with infusion of leu-enkephalin (m/z (2+) = 556.2771) every 30 s. For peptide identification, the mass spectrometer was set to collect data in MS^E^, ESI+ mode instead. The peptides were identified from triplicate analyses of 12 μM muPA^16-243^, and data were analysed using PLGS 2.5 (Waters Corporation). Peptides masses were identified using a minimum number of 250 ion counts for low energy peptides and 50 ion counts for their fragment ions. The following cut-offs were used to filter peptide sequences matches: minimum products per amino acid of 0.2, minimum score of 8, maximum MH+ error of 3 ppm, a retention time RSD of 5%, and the peptides had to be present in two out of the three identification runs. The peptides identified in PLGS were analysed in DynamiX 3.0 (Waters Corporation). The relative deuterium uptake for each peptide was calculated by comparing centroids of the mass envelopes of the deuterated samples with the undeuterated controls (timepoint 0 min).

### Chromogenic substrate assays

For evaluating the effect of FabmU1 and FabmU3 on the catalytic activity of muPA^16-243^, various concentrations of FabmU1 (500–0 nM) or FabmU3 (20–0 nM) were incubated with 1 nM muPA^16-243^ for 15 min at 37°C in a buffer containing 10 mM HEPES pH 7.4, 140 mM NaCl and 0.1% BSA. The chromogenic substrate Pyro-Glu-Gly-Arg-pNa (CS-61(44), Aniara, USA) was added to a final concentration of 750 μM, and the initial velocities was monitored for 1 h at 37°C at an absorbance of 405nm in a microplate reader. For the inhibition of muPA^16-243^ catalytic activity by mU1, the apparent inhibitory constant ($K_{i}^{app}$) was calculated as described in [16].

Kinetic analysis of plasminogen activation by full-length muPA was performed using an indirect chromogenic assay. Plasminogen (24–0 μM), purified from outdated human plasma [21], was incubated with full-length muPA or full-length muPA alanine mutants (1 nM) for 30 min at 37°C. Full-length muPA activity was quenched by adding the muPA competitive inhibitor mupain-1-IG (10 μM) [22]. The activity of muPA-generated plasmin was evaluated by adding the chromogenic substrate S-2251 (0.5 mM), and the initial velocities were monitored for 1 h at 37°C at an absorbance of 405nm in a microplate reader. *K*_*M*_, and *k*_*cat*_ was determined by fitting the data using standard Michaelis-Menten kinetics. The experimental procedure was limited to a maximum plasminogen concentration of 24 μM, as a higher plasminogen concentration resulted in irreversible protein aggregation.

## Results

**Fig 1:**
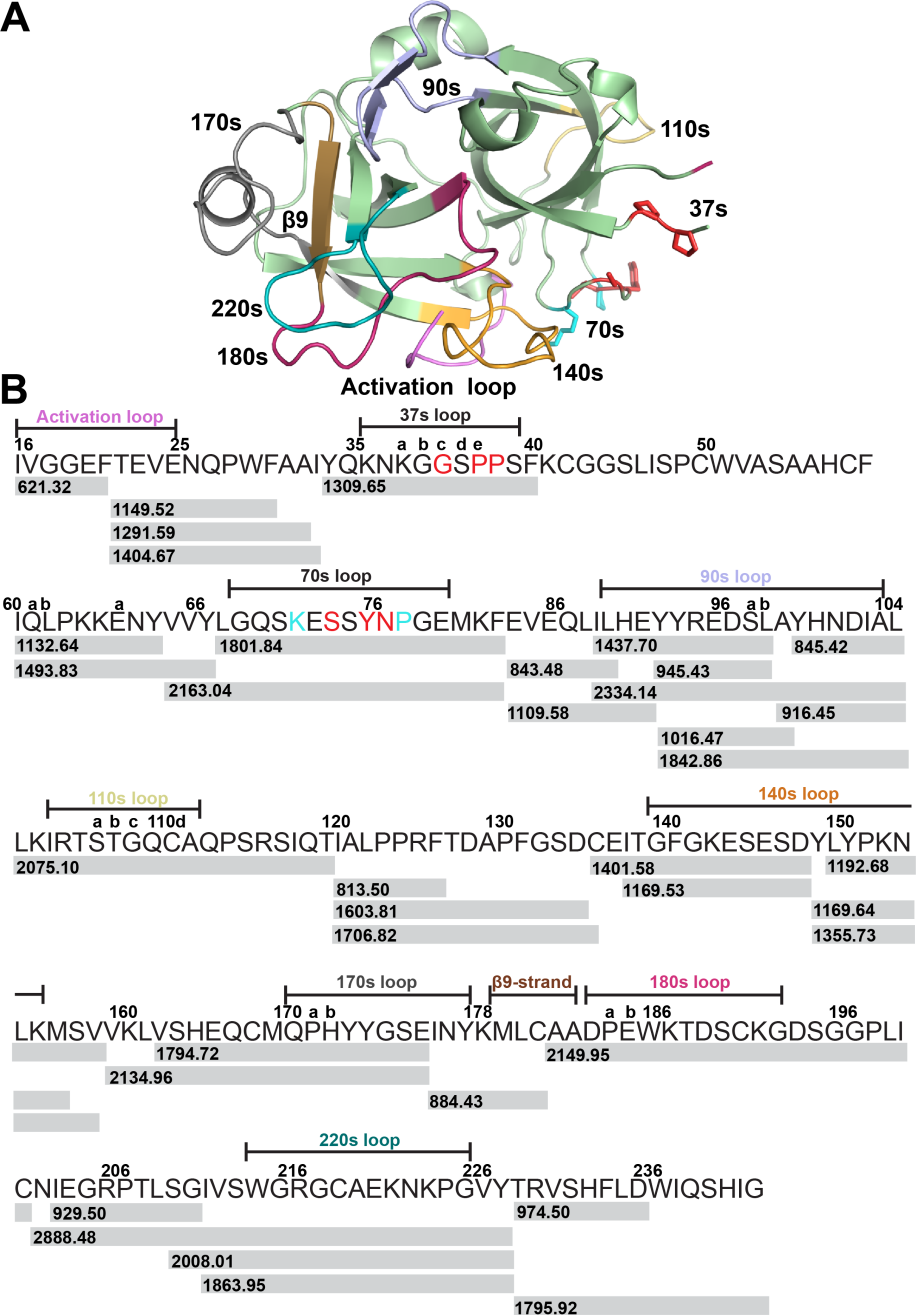
HDXMS coverage map. **(A)** The X-ray crystal structure of the catalytic domain of muPA^16-243^ in its active conformation (PDB ID 5LHR). Important regions in muPA^16-243^ are highlighted by colors (A); light-pink for the activation loop, light blue for the 90s loop, yellow for the 110s loop, orange for the 140s loop, gray for the 170s loop, brown for the β9-strand, red for the 180s loop and teal for the 220s loop. Residues in the 37s and 70s that are in common for the mU1 and mU3 binding epitopes are highlighted in red and shown as sticks. Resides that are unique to the mU1 binding epitope are highlighted in cyan and shown as sticks. (**B)** The HDXMS coverage map. Gray bars underneath the muPA^16-243^ sequence show the pepsin-generated peptides. Peptide masses for each peptide are indicated on the bar.

HDXMS analyses were performed with the catalytic serine protease domain of muPA (muPA^16-243^) in the following states: ligand free muPA^16-243^ (*apo*-muPA^16-243^), EGR-cmk-bound muPA^16-243^, mU1-bound muPA^16-243^ and mU3-bound muPA^16-243^. We have recently reported the HDXMS uptake plots for selected peptides in the muPA^16-243^ and EGR-cmk bound muPA^16-243^ variants [15]. Here, we perform the HDXMS experiments again in order to provide the complete peptide coverage map and corresponding uptake plots of all pepsin-generated peptides in muPA^16-243^ and EGR-cmk bound muPA^16-243^. The proteins or complexes were diluted into buffered D_2_O and the resulting mass increase due to incorporation of deuterium was monitored as a function of time (0, 0.5, 1, 2, 5 min). Deuterium incorporation was localized to various regions of muPA^16-243^ by mass analysis of peptides produced by pepsin proteolysis, resulting in the identification of 36 overlapping peptides that together cover 92% of the muPA^16-243^ sequence (Fig 1A).

**Fig 2:**
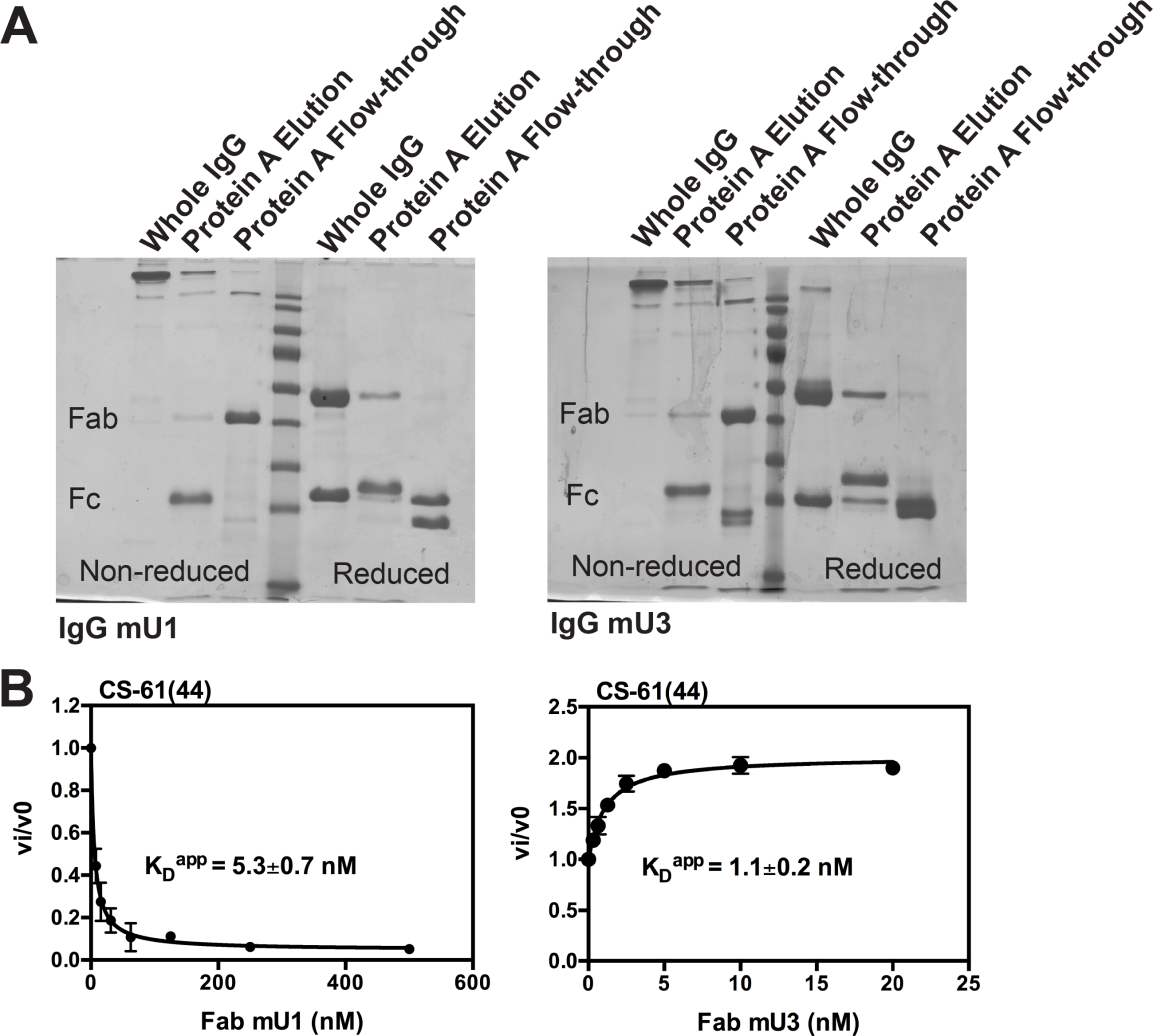
Generation and characterization of FabmU1 and FabmU3. **(A)** SDS-PAGE analysis of the whole IgG and papain-cleaved IgG after protein A affinity chromatography. **(B)** The effect of FabmU1 (left) and FabmU3 (right) to the catalytic activity of muPA^16-243^ towards the small chromogenic substrate Glu-Gly-Arg-pNA (CS-61(44)). Error bars, s.d. (n = 3 independent measurements).

To simplify the HDXMS analysis, we generated Fab fragments of the monoclonal antibodies mU1 and mU3 (Fig 2A). In agreement with previously published results with the intact full-length monoclonal antibodies, FabmU1 inhibited muPA^16-243^ hydrolysis of a small chromogenic substrate, whereas FabmU3 stimulated the activity of muPA^16-243^ by 2-fold (Fig 2B). We have previously determined the binding epitopes of mU1 and mU3 by alanine scanning mutagenesis. Whereas residues Gly37c, Pro37e, Pro38, Ser74, Tyr76 and Asn77 in muPA were determined to be important for the binding of both mU1 and mU3, residues Lys72 and Pro78 were only important for the binding of mU1 (Fig 1A).

### Changes in amide exchange of muPA^16-243^ upon binding of EGR-cmk

**Fig 3:**
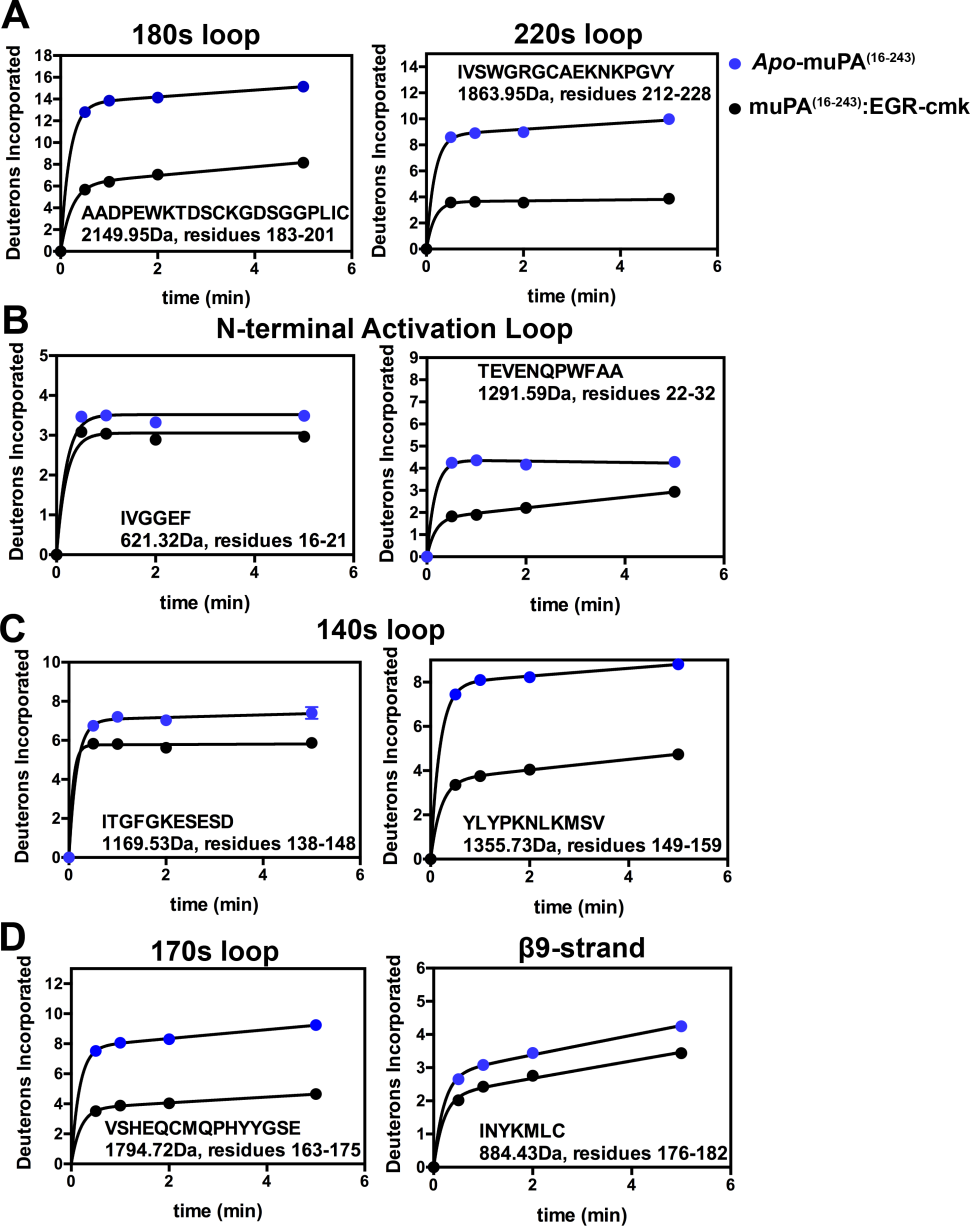
Relative deuterium uptake plots for peptides in *apo*-muPA^16-243^ versus EGR-cmk-bound *muPA*^*16-243*^. The figure shows uptake plots from peptides covering **(A)** the active site regions 180s and 220s loops, **(B)** the activation loop, **(C)** the 140s loop, **(D)** the β9-strand and the 170s loop. Peptide sequence, masses and residues numbers are shown for each peptide. The Y-axis is scaled to show the theoretical maximum deuterium uptake of the corresponding peptide. Error bars, s.d. (n = 3 independent measurements).

We have previously shown how the binding of EGR-cmk to the active site of muPA^16-243^ decreases deuterium incorporation in peptides containing amino acids that are within contact distance of (<4Å) of the EGR-cmk molecule [15]. This includes residues in the 180s and 220s loops. In agreement with this result, we found that the binding of EGR-cmk significantly reduces deuterium uptake in four peptides that covers the 180s and 220s loops (Fig 3A). One peptide corresponded to the 180s loop (residues 183–201, MH^+^ 2149.95Da), and three overlapping peptides corresponded to the 220s loop (residues 202–228, MH^+^ 2888.48Da; residues 210–228, MH^+^ 2008.00Da; residues 212–228, MH^+^ 1863.95Da). Fig 3A shows the deuterium uptake curves for residues 183–201 and residues 210–228 (the remaining peptides are shown in S1 Fig). Six peptides corresponded to 90s loop (residues 89-97b, MH+ 1437.70Da; residues 93-97b, MH+ 945.43Da; residues 93–98, MH+ 1016.46Da; residues 93–105, MH+ 1842.86Da; residues 98–105, MH+ 916.45Da; residues 99–105, MH+ 845.41Da). Somewhat surprisingly no differences in deuterium incorporation were observed in any of the 90s loop peptides despite the fact that residues Leu97b and Tyr99 have been shown to be important in substrate recognition [23] (S1 Fig).

Four peptides corresponding to the new N-terminal sequence produced by plasmin were identified by the analysis. Although all amino acids in the N-terminal sequence are more than 4 Å away from the EGR-cmk molecule, decreased deuterium incorporation upon EGR-cmk binding was observed in all four peptides. Whereas the peptide covering the N-terminal Ile-16 (residues 16–21, MH^+^ 621.32Da) only showed a slight reduction in deuterium incorporation, the decreased deuterium incorporation in the three peptides covering the following stretch of amino acids was more pronounced (Fig 3B) (residues 22–30, MH^+^ 1149.52; residues 22–32, MH^+^ 1291.59Da; residues 22–33, MH^+^ 1404.67Da). No other significant changes in amide exchange were observed upon EGR-cmk binding in the reminder of the N-terminal β-barrel (S1 Fig).

Five peptides corresponded to the 140s loop (residues 136–148, MH^+^ 1401.58Da; residues 138–148, MH^+^ 1169.53Da; residues 149–157, MH^+^ 1169.63Da; residues 149–159, MH^+^ 1355.73Da; residues 150–159, MH^+^ 1192.67Da). The 140s loop is known as a very flexible loop in trypsin-like serine proteases, and it has been shown that binding of active site ligands allosterically reduces the conformational flexibility of this loop [24]. In good agreement with this notion, our results revealed that all five peptides covering the 140s loop showed decreased deuterium incorporation upon binding of EGR-cmk (Figs 3C and S1).

We have previously shown that binding of EGR-cmk to the active site of muPA^16-243^ allosterically affects the conformational flexibility of the β9-strand and 170s loop [16]. In agreement with our previous findings, our HDXMS results showed that the binding of EGR-cmk reduced deuterium incorporation in both peptides covering the 170s loop (residues 160–175, MH^+^ 160–175; residues 163–175, MH^+^ 1794.72Da) and in the single peptide covering the β9-strand (residues 176–182, MH^+^ 884.43) (Figs 3D and S1).

### Changes in amide exchange of the 37s and 70s loops in muPA^16-243^ upon binding of FabmU1 and FabmU3

**Fig 4:**
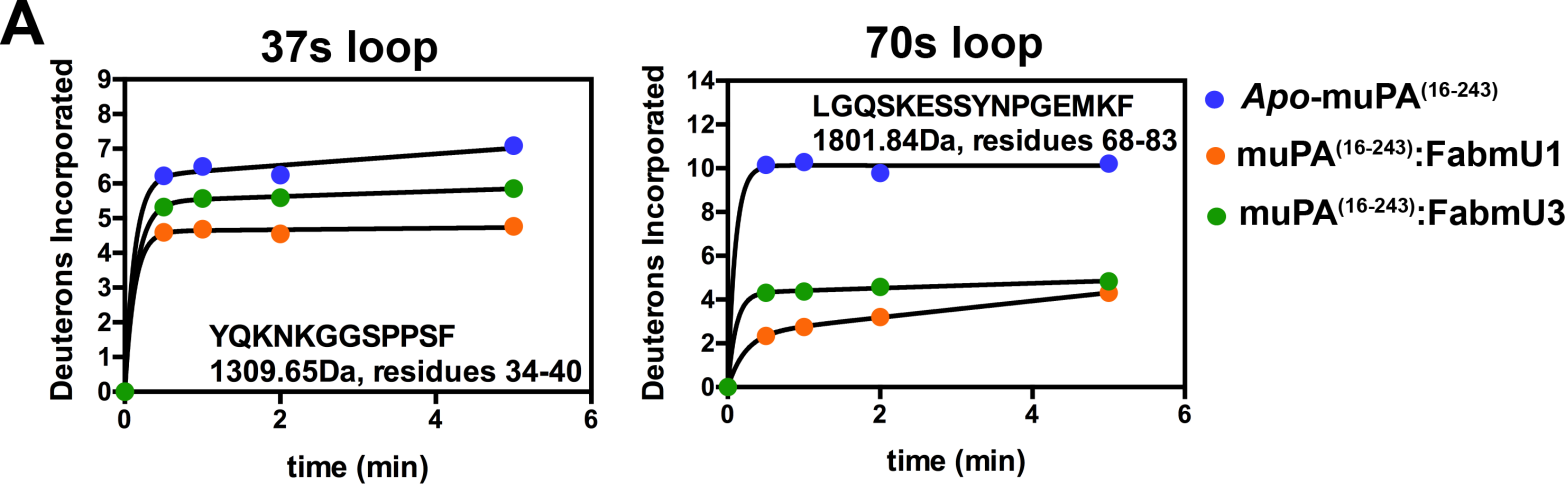
Relative deuterium uptake plots for peptides in *apo*- muPA^16-243^ versus FabmU1:muPA^16-243^ and FabmU3:muPA^16-243^. The figure shows uptake plots from peptides covering the 37s and 70s. Peptide sequence, masses and residues numbers are shown for each peptide. The Y-axis is scaled to show the theoretical maximum deuterium uptake of the corresponding peptide. Error bars, s.d. (n = 3 independent measurements).

Given the high sequence coverage and extensive changes throughout muPA^16-243^ induced by EGR-cmk binding, HDXMS was expected to reveal the differences in conformational states accounting for the opposite functional effect of mU1 and mU3 on the catalytic activity of muPA^16-243^. When compared with *apo*- muPA^16-243^ the HDXMS results revealed decreased deuterium incorporation in three peptides covering the FabmU1 and FabmU3 binding epitopes in the 37s and 70s loops (residues 34–40, MH^+^ 1309.65Da; residues 65–83, MH^+^ 2163.04Da; residues 68–83, MH^+^ 1801.84Da) (Figs 4 and S2). The decreased exchange at the binding site is likely due to a combination of decreased surface solvent accessibility due to formation of the Fab binding interface as well as perhaps additional localized dampening of loop dynamics. The dampening effect was observed by a reduced exchange in the 110s loop (residues 106–120, MH^+^ 2075.10Da) (S2 Fig), which was not identified as a part of the mU1 and mU3 binding epitopes in the alanine scanning mutagenesis [16, 17].

### Difference in amide exchange of the muPA^16-243^ active site region upon binding of FabmU1 or FabmU3

**Fig 5:**
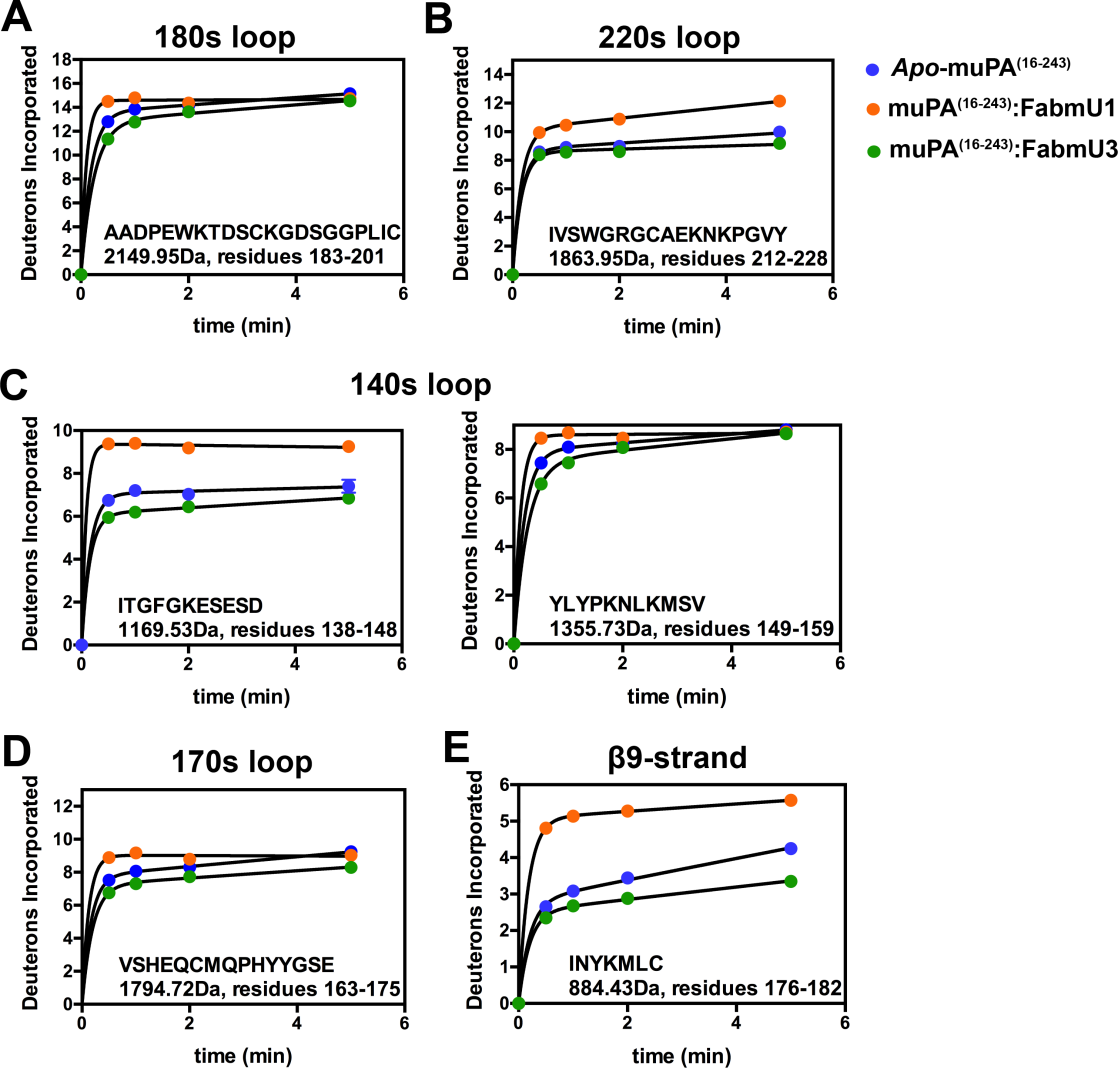
Relative deuterium uptake plots for peptides in *apo*-muPA^16-243^ versus FabmU1:muPA and FabmU3:muPA. The figure shows uptake plots from peptides covering **(A)** the 180s loop, **(B)** the 220s loop, **(C)** the 140s loop, **(D)** the 170s loop and the β9-strand. Peptide sequence, masses and residues numbers are shown for each peptide. The Y-axis is scaled to show the theoretical maximum deuterium uptake of the corresponding peptide. Error bars, s.d. (n = 3 independent measurements).

When compared to *apo*- muPA^16-243^, the binding of FabmU1 to muPA^16-243^ caused increased deuterium incorporation in several regions of muPA^16-243^ whereas FabmU3 caused slightly decreased exchange in many of these same regions. The peptides corresponding to the 180s and 140s loops are examples of such opposing effects of the two Fab fragments (Fig 5A and 5B). Interestingly, the peptide covering the 180s loop harbors the catalytic Ser195 and residues forming the S1 specificity pocket (Asp189) and the oxyanion hole (Gly193 and Ser195). In addition, FabmU1 caused an increase in amide exchange of peptides covering several loops surrounding the muPA^16-243^ active site, including five peptides in the 140s loop (Figs 5B and S2), two peptides in the 170s loop (Figs 5C and S2) and in the single peptide covering the β9-strand (Fig 5D). Oppositely to FabmU1, FabmU3 caused a decreased deuterium uptake in the same peptides (Fig 5B, 5C and 5D). FabmU1 binding also caused increased deuterium incorporation in both peptides corresponding to the 220s loop, whereas FabmU3 had no significant effect on this region (Figs 5E and S2).

The binding of FabmU1 and FabmU3 resulted in complex differences in amide exchange in different regions of the 90s loop in muPA^16-243^. Binding of both FabmU1 and FabmU3 caused decreased deuterium incorporation in the peptide corresponding to residues 93–98 of the 90s loop (MH^+^ 1016.46Da) (S2 Fig). However, FabmU1 and FabmU3 had opposite effects on deuterium incorporation in the peptide corresponding to residues 93–105 (MH^+^ 1842.86Da) (S2 Fig).

### A possible role of the muPA^16-243^ 70s and 140s loops in plasminogen activation

The 140s loop in the uPA homologue tissue-type plasminogen activator (tPA) has previously been identified as an important exosite for recognition of the physiological uPA substrate, plasminogen [25]. As the 70s loop is in the vicinity of the 140s loop, and since our HDXMS results showed that binding of FabmU1 or FabmU3 to the 70s loop affected the conformational flexibility of the 140s loop differently, we now hypothesized that the 70s loop may be important for the recognition of plasminogen either directly or indirectly by controlling the conformational flexibility of the 140s loop. To investigate this hypothesis, we generated a panel of full-length muPA alanine substitution mutants and evaluated their kinetic parameters for plasminogen activation and hydrolysis of the small chromogenic substrate Glu-Gly-Arg-pNA (Tables 1 and 2). The measured *K*_*M*_ and *k*_*cat*_ for plasminogen activation by full-length muPA were 9.8 μM and 3.5 s^-1^ respectively. Mutation of Lys72, Tyr76, Asn77 and Pro78 to Ala caused small decreases in *K*_*M*_ for plasminogen activation and a slight but insignificant increase in the *k*_*cat*_. Mutation of Glu73 and Ser74 to Ala strongly affected plasminogen activation by causing a more than 2-fold increase in *K*_*M*_ (24.6 μM and 22 μM respectively). However, a significant decrease in *k*_*cat*_ was only observed for the Glu73 to Ala mutation but not for the Ser74 to Ala mutation (Table 1). The most dramatic effect on plasminogen activation was observed upon mutation of Tyr149 to Ala, which caused a 60-fold decrease in *k*_*cat*_ without affecting *K*_*M*_ (Table 1). Interestingly, the Tyr149 to Ala mutation showed opposite effects on the kinetics of hydrolysis of the small chromogenic substrate, for which an increase in *k*_*cat*_ and a decrease in *K*_*M*_ were observed (Table 2). Alanine substitutions in the 70s loop largely did not affect *K*_*M*_ and *k*_*cat*_ for the hydrolysis of the small chromogenic substrate. In summary, these results show that residues in the 70s loop are important for the recognition of plasminogen, but not for cleavage of small chromogenic substrates.

**Table 1:** Kinetic analysis for plasminogen activation by full-length muPA and variants.

	K_M_ (μM)	*k*_*cat*_ (s^-1^)	*k*_*cat*_/K_M_ (μM^-1^ s^-1^)
**Full-length muPA**	9.8 ± 1.1	3.5 ± 0.9	0.4 ± 0.1
**Full-length muPA K72A**	3.1 ± 0.9*	5.0 ± 1.9	1.6 ± 0.1*
**Full-length muPA E73A**^**¶**^	24.6 ± 2.8*	1.3 ± 0.1*	0.05 ± 0.01*
**Full-length muPA S74A**^**¶**^	22.0 ± 3.8*	3.5 ± 0.6	0.16 ± 0.01*
**Full-length muPA Y76A**	5.0 ± 2.4*	5.1 ± 1.5	1.2 ± 0.7*
**Full-length muPA N77A**	5.1 ± 0.8*	4.8 ± 0.8	1.0 ± 0.01*
**Full-length muPA P78A**	3.9 ± 1.1*	5.2 ± 1.2	1.3 ± 0.1*
**Full-length muPA Y149A**	12.0 ± 2.7	0.06 ± 0.04 *	0.0053 ± 0.0045*

*Significantly different from the corresponding value determined for full-length muPA (p < 0.05)–student’s t-test.

^**¶**^ The K_M_ is equal to the highest plasminogen concentration used (24 μM).

**Table 2:** Kinetic analysis for hydrolysis of a chromogenic substrate CS-61(44) by full-length muPA and variants.

	K_M_ (μM) S-2444	*k*_*cat*_ (s^-1^) S-2444	*k*_*cat*_/K_M_ (μM^-1^ s^-1^)
**Full-length muPA**	2600 ± 200	10.9 ± 2.3	0.004 ± 0.001
**Full-length muPA K72A**	2600 ± 200	6.6 ± 1.4*	0.003 ± 0.001
**Full-length muPA E73A**	1860 ± 270*	11.7 ± 1.8	0.006 ± 0.001
**Full-length muPA S74A**	2900 ± 100	12.8 ± 4.3	0.004 ± 0.002
**Full-length muPA Y76A**	2600 ± 100	9.0 ± 1.2	0.004 ± 0.001
**Full-length muPA N77A**	3800 ± 115*	8.6 ± 1.8	0.002 ± 0.001
**Full-length muPA P78A**	2500 ± 100	9.7 ± 1.6	0.004 ± 0.001
**Full-length muPA Y149A**	1400 ± 320*	39 ± 10*	0.03 ± 0.01*

*Significantly different from the corresponding value determined for full-length muPA (p < 0.05)–student’s t-test.

## Discussion

**Fig 6:**
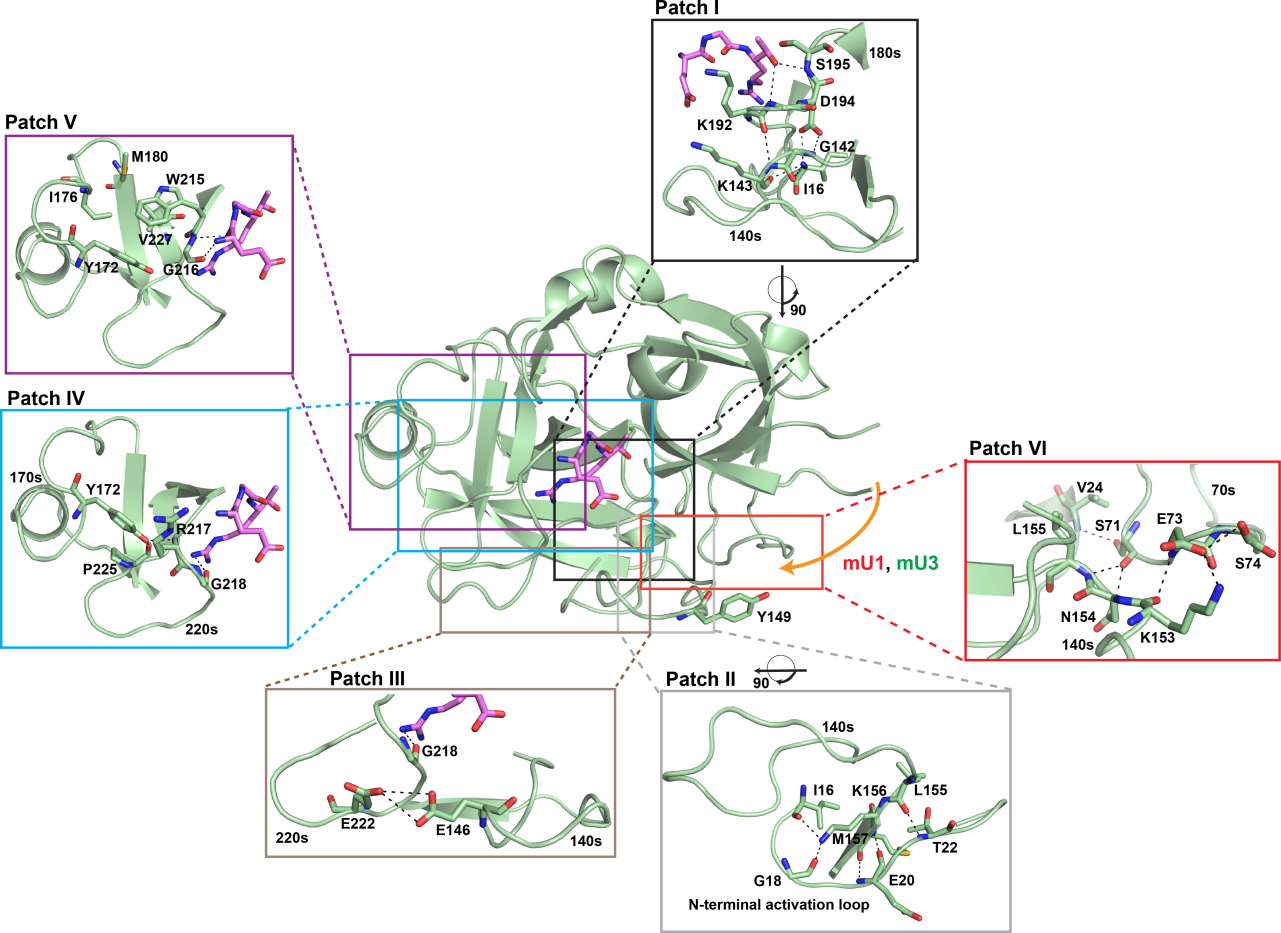
The allosteric pathway in muPA. Structural representation of muPA^16-243^ in complex with EGR-cmk (The model was prepared using PDB ID 5LHQ and 5LHR). The mU1 and mU3 binding site in the 37s and 70s loops is indicated by an orange arrow. Regions identified by the HDXMS analysis to be affected differently by FabmU1 and FabmU3 are highlighted by colored boxes; black: 180s loop of the active-site region, grey: the N-terminal base of the 140s loop, red: the C-terminal base of the 140s loop, brown: the 220s loop, blue: the β9-strand, purple: the 170s loop. The colored boxes are also displayed as zooms of Patch I: The interaction network between the N-terminal base of the 140s loop and residues of the oxyanion hole; Patch II: The interaction network between the C-terminal base of the 140s loop and residues of the N-terminal activation loop; Patch III: The interactions between the 140s loop and the 220s loop; Patch IV: The interaction between the 170s loop and the 220s loop; Patch V: The hydrophobic interaction between the 170s loop and the 220s loop. Patch VI The 70s/140s interaction network. In all figures interacting residues are shown as sticks and black dashed lines represent potential hydrogen bonds. The figure was prepared using PyMOL version 1.7.4.0.

When compared to *apo*- muPA^16-243^, EGR-cmk-bound muPA^16-243^ showed decreased amide exchange in regions corresponding to the N-terminal activation loop, the 140s, the 180s, and the 220s loops. The four loops are interconnected through three patches of polar interaction networks (patch I, II and III) (Fig 6). In muPA^16-243^, patch I interconnects the N-terminal base of the 140s loop with the 180s loop (Fig 6). Residues Lys192 and Asp194, which is adjacent to the oxyanion hole residues (Gly193 and Ser195), are in direct contact with the residues Gly142 and Lys143 of the 140s loop. Patch II interconnects the C-terminal base of the 140s loop to the N-terminal activation loop. Residues Leu155, Lys156 and Met157 of the 140s loop are in direct contact with residues Ile16, Gly18, Glu20 and Thr22 in the N-terminal sequence (Fig 6). Patch III interconnects the 140s loop and the 220s loop. Glu146 contacts Glu222, which is in vicinity of residues of the S1 specificity pocket (Gly218) (Fig 6). Our HDXMS results clearly show that the N-terminal sequence, the 140s, the 180s, and the 220s loops is highly dynamic in *apo*- muPA^16-243^. Binding of EGR-cmk, however, dampens exchange throughout the loops presumably by strengthening the interconnectivity of stabilizing interactions in patches I, II and III. Importantly, our HDXMS data revealed a relatively high amide exchange level (71%) in the peptide covering the N-terminal Ile16 suggesting that Ile-16 may be mostly solvent-exposed. This observation is in good agreement with the notion that *apo*- muPA^16-243^ crystallizes in a conformation with a solvent-exposed Ile16 [15].

EGR-cmk-bound muPA^16-243^ also showed a decreased amide exchange level in the β9-strand and 170s loops when compare to *apo*- muPA^16-243^. We have recently showed that the 170s loop in muPA exists in equilibrium between open and closed conformations [15]. The decreased amide exchange in the 170s loop in EGR-cmk-bound muPA^16-243^ shows that EGR-cmk stabilizes the closed conformation. Since the *β*9-stand is tucked away underneath the 170s loop, the closed 170s loop conformation is also expected to protect the *β*9-stand from amide exchange. The active site region of muPA is interconnected to the 170s loop through two patches of polar interaction networks (patch IV and V). In patch IV Tyr172 of the 170s loop forms hydrogen bonds with the backbone N-atom of Arg217 and the carbonyl O-atom of Pro-225 from the 220s loop (Fig 6). Since Arg217 is part of the S3 substrate binding pocket and positioned next to Gly218, which interacts directly with the Arg-moiety of EGR-cmk, it is reasonable to assume that EGR-cmk stabilizes the closed 170s loop conformation by stabilizing the Tyr172-Arg217 interaction. In patch V, the stabilizing effects of EGR-cmk on the 170s loop may propagate through Trp215. Trp215 is buried in a hydrophobic pocket created by residues in the 170s and 220s loops (Tyr172, Ile176, Met180 and Val227) and the *β*12-strand (Val227) (Fig 6). Since the Trp215 is involved in substrate recognition together with Gly216 as a part of the S2 binding pocket, the stabilizing effect of EGR-cmk to Trp215 may secure its position in the hydrophobic pocket further stabilizing the 170s loop in the closed conformation.

In X-ray crystal structures of trypsin-like serine proteases in their active conformation, residues in the 70s loop are forming multiple hydrogen bonds with residues at the C-terminal stem of the 140s loop. In muPA the carbonyl group of Ser-71 is within hydrogen bonding distance of the backbone N-atom of Leu-155 and the side chain N-atom of Asn-154. Furthermore, the backbone N-atom of Glu-73 contacts the backbone carbonyl O-atom of Lys-153, whereas the side chain Oε1 atom of Glu-73 contacts the side chain N-atom of Lys-153 (Fig 6). In addition, the OH-group of Ser74 stabilizes the side chain of Glu73 in the groove between the 70s and 140s loop by forming a hydrogen bond to the Oε1 moiety of Glu73. Although the exact binding mechanism of FabmU1 and FabmU3 must await structural characterization, it is interesting to surmise that the hydrogen bonds between the 70s and 140s loops (patch VI) may be destabilized in the FabmU1-bound muPA^16-243^ conformation, but stabilized in FabmU3-bound muPA^16-243^. This hypothesis readily explains the different functional effects of mU1 and mU3 to the muPA^16-243^ activity, as the 140s loop is interconnected with the active site region through patch I, II and III. Our HDXMS data strongly supports this view by showing that binding of FabmU1 to muPA^16-243^ caused an increase in amide exchange in the 140s, 170s, 180s, 220s loops and the *β*9-stand, whereas FabmU3 binding to muPA^16-243^ caused decreased amide exchange. Thus, mU1 stabilizes a highly dynamic, inactive muPA^16-243^ conformation, whereas mU3 stabilizes a less dynamic, active muPA^16-243^ conformation.

The loop corresponding to the 70s loop in the coagulation protease, thrombin, is part of the regulatory anion binding exosite 1 (ABE1). Binding of ligands, cofactors and substrates to ABE1 allosterically regulates the activity and function of the active site in thrombin [10, 26]. The 70s loop is also known as an activity regulatory divalent cation binding site in some trypsin-like serine protease including the coagulation factors VIIa and X, trypsin and the kallikrein-related protease 4 [27–31]. Moreover, the 70s loop has been shown to participate in allosteric networks in trypsin-like serine proteases by interconnecting the 70s loop to the N-terminal activation loop, the 90s loop and to the 140s loop [32–34].

Although the plasminogen-activation system has been thoroughly studied for more than 50 years, the mechanism of plasminogen activation has remained largely obscure. This is due to the lack of structural information about the plasminogen-plasminogen activator (tPA or uPA) complexes. Here, we went in another direction and combined the information from the HDXMS with a kinetic analysis to propose a model for muPA-mediated plasminogen activation. In agreement with previous results in which the binding interface between tPA and plasminogen was mapped, we found that the 140s loop is important in plasminogen activation by muPA, finding that the Tyr149 to Ala mutation caused a marked decrease in *k*_*cat*_. Interestingly, the side-chain moiety of Tyr149 is solvent-exposed and positioned distantly from the surrounding surface-exposed loops in muPA (Fig 6). The observed change in *k*_*cat*_ but not *K*_*M*_ suggests that Tyr149 play an important role in positioning of plasminogen as a substrate for efficient cleavage by muPA. Our analysis further revealed an important role of the 70s loop in muPA for plasminogen activation, as mutation of Glu73 and Ser74 to Ala caused an increase in *K*_*M*_. Since the *K*_*M*_ for hydrolysis of the small chromogenic substrate remained unchanged for the Glu73 and Ser74 to Ala mutants, our data suggests that the 70s loop forms an important secondary exosite of interaction between muPA and plasminogen. Interestingly, the HDXMS data showed that binding of ligands, such as mU1 and mU3, to the 37s and 70s loops may stabilize more or less dynamic muPA^16-243^ conformations. Thus, the HDXMS and kinetic results strongly supports a model of plasminogen activation in which plasminogen primes its own activation through binding to the 70s and/or 140s loops thereby stabilizing muPA in a less dynamic active conformation.

The observation that mU1 stabilizes an inactive muPA^16-243^ conformation, which by many criteria is similar to a muPA conformation recently described in a X-ray crystal structure of *apo*- muPA^16-243^ is of significant importance since mU1 has been shown to inhibit muPA dependent processes *in vivo* such as reduction of atherosclerosis in mice [15, 18, 19]. Collectively, these results strongly suggest that a muPA conformation with a highly distorted C-terminal *β*-barrel may exist *in vivo* under normal or pathological conditions. Thus, targeting inactive uPA conformations with allosteric agents such as mU1 represents an attractive strategy for intervention with the pathophysiological activities of uPA.

## Supporting information

S1 Fig Relative deuterium uptake. Uptake plots for the 28 peptides not shown in the main text covering *apo*-muPA^16-243^ (blue dots) and muPA^16-243^:EGR-cmk (black dots). Peptide sequence, masses and residues numbers are shown for each peptide. The Y-axis is scaled to show the theoretical maximum deuterium uptake of the corresponding peptide. Error bars, s.d. (n = 3 independent measurements).
(TIF)

S2 Fig Relative deuterium uptake. Uptake plots for the 28 peptides not shown in the main text covering *apo*-muPA^16-243^ (blue dots), muPA^16-243^:FabmU1 (orange dots) and muPA^16-243^:FabmU3 (green dots). Peptide sequence, masses and residues numbers are shown for each peptide. The Y-axis is scaled to show the theoretical maximum deuterium uptake of the corresponding peptide. Error bars, s.d. (n = 3 independent measurements).
(TIF)
