# Supplementary figures and images for: Ligand binding modulates the structural dynamics and activity of urokinase-type plasminogen activator: A possible mechanism of plasminogen activation
Source: PLoS One. 2018 Feb 8;13(2):e0192661. doi: 10.1371/journal.pone.0192661 (PMC5805342; doi:10.1371/journal.pone.0192661)

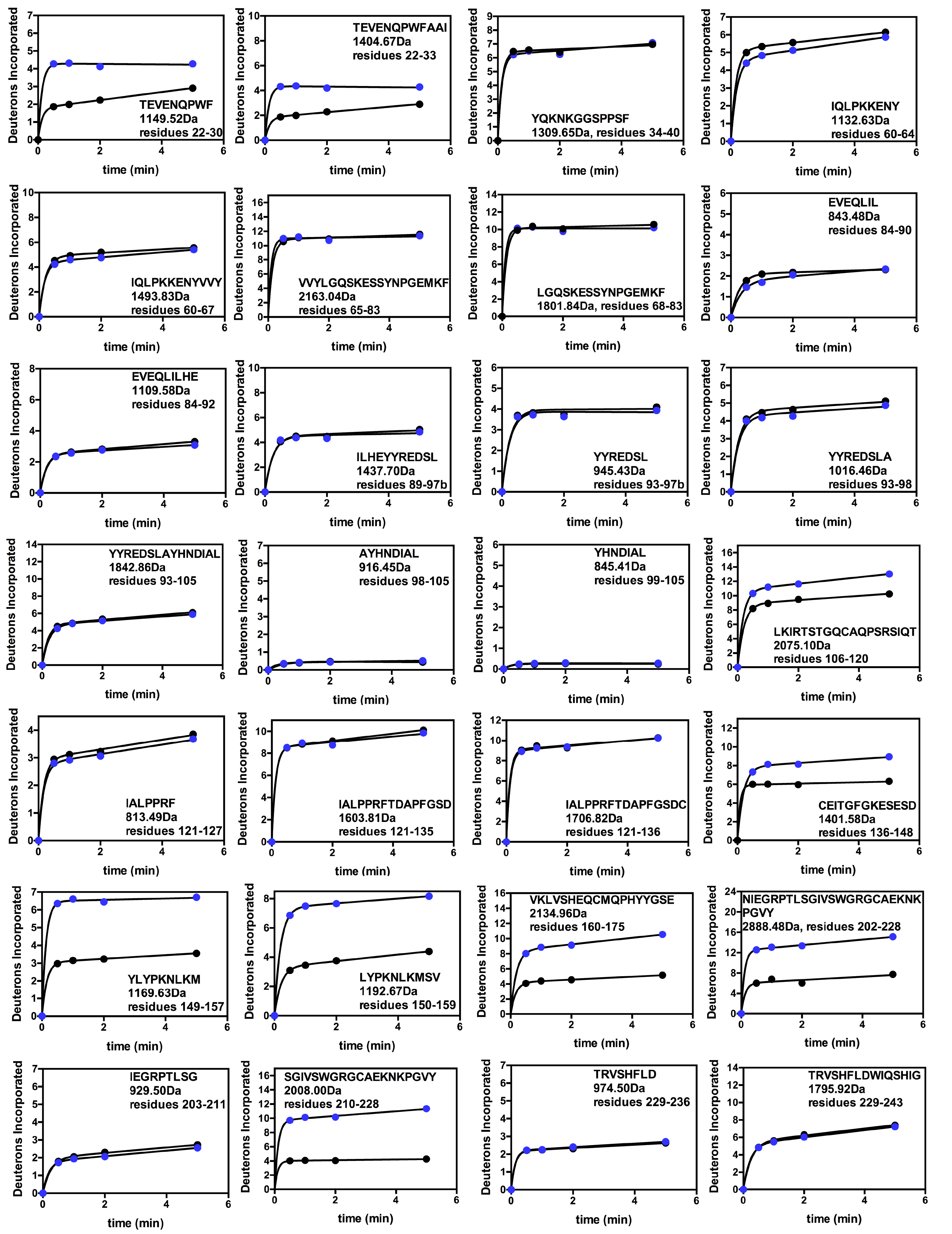

Supplement: S1 Fig — Uptake plots for the 28 peptides not shown in the main text covering apo-muPA16-243 (blue dots) and muPA16-243:EGR-cmk (black dots). Peptide sequence, masses and residues numbers are shown for each peptide. The Y-axis is scaled to show the theoretical maximum deuterium uptake of the corresponding peptide. Error bars, s.d. (n = 3 independent measurements). (TIF) [file pone.0192661.s001.tif]

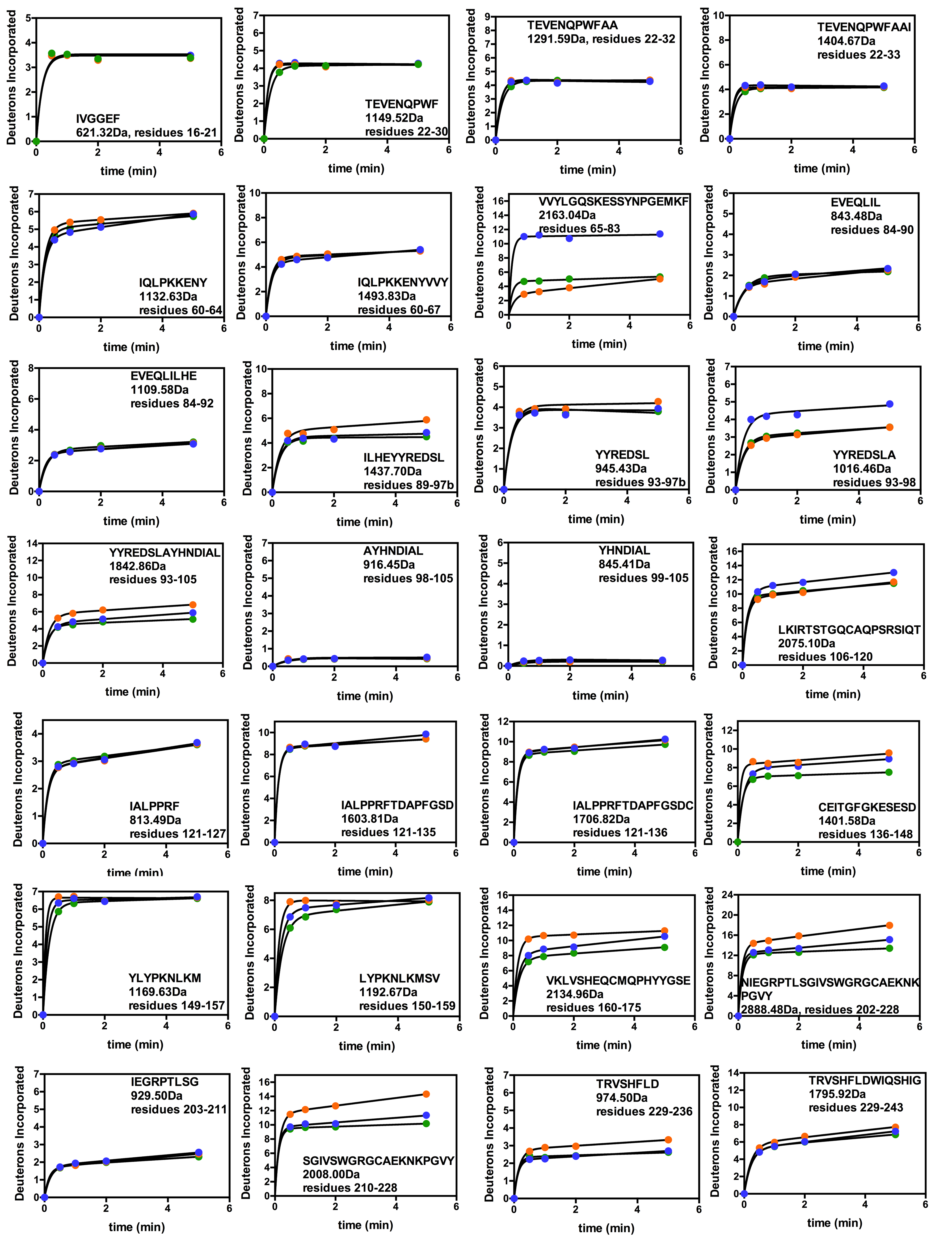

Supplement: S2 Fig — Uptake plots for the 28 peptides not shown in the main text covering apo-muPA16-243 (blue dots), muPA16-243:FabmU1 (orange dots) and muPA16-243:FabmU3 (green dots). Peptide sequence, masses and residues numbers are shown for each peptide. The Y-axis is scaled to show the theoretical maximum deuterium uptake of the corresponding peptide. Error bars, s.d. (n = 3 independent measurements). (TIF) [file pone.0192661.s002.tif]
